# Social and economic development and pregnancy mental health: secondary analyses of data from rural Vietnam

**DOI:** 10.1186/s12889-020-09067-9

**Published:** 2020-06-26

**Authors:** Ruby Stocker, Trang Nguyen, Thach Tran, Ha Tran, Tuan Tran, Sarah Hanieh, Beverley-Ann Biggs, Jane Fisher

**Affiliations:** 1 Global and Women’s Health, School of Public Health and Preventive Medicine, Monash University, Level 4, 553 St Kilda Rd, Melbourne, Victoria 3004 Australia; 2 Research and Training Centre for Community Development, No 39 Lane 255, Vong Street, Hai Ba Trung District, Hanoi, Vietnam; 3 Department of Medicine and Victorian Infectious Diseases Services at the Doherty Institute, University of Melbourne, 792 Elizabeth Street, Melbourne, 3000 Australia

**Keywords:** Mental health, Depression, Anxiety, Pregnancy, Women’s health, Vietnam, Socioeconomic factors, Social change, Economic development

## Abstract

**Background:**

This study aimed to establish whether changes in the socioeconomic context were associated with changes in population-level antenatal mental health indicators in Vietnam.

**Methods:**

Social, economic and public policies introduced in Vietnam (1986–2010) were mapped. Secondary analyses of data from two cross-sectional community-based studies conducted in 2006 (*n* = 134) and 2010 (*n* = 419), involving women who were ≥ 28 weeks pregnant were completed. Data for these two studies had been collected in structured individual face-to-face interviews, and included indicators of antenatal mental health (mean Edinburgh Postnatal Depression Scale Vietnam-validation (EPDS-V) score), intimate partner relationships (Intimate Bonds Measure Vietnam-validation) and sociodemographic characteristics. Socioeconomic characteristics and mean EPDS-V scores in the two study years were compared and mediation analyses were used to establish whether indicators of social and economic development mediated differences in EPDS-V scores.

**Results:**

Major policy initiatives for poverty reduction, hunger eradication and making domestic violence a crime were implemented between 2006 and 2010. Characteristics and circumstances of pregnant women in Ha Nam improved significantly. Mean EPDS-V score was lower in 2010, indicating better population-level antenatal mental health. Household wealth and intimate partner controlling behaviours mediated the difference in EPDS-V scores between 2006 and 2010.

**Conclusions:**

Changes in the socioeconomic and political context, particularly through policies to improve household wealth and reduce domestic violence, appear to influence women’s lives and population-level antenatal mental health. Cross-sectoral policies that reduce social risk factors may be a powerful mechanism to improve antenatal mental health at a population level.

## Background

From conception to the age of three development in all domains is rapid, and young children are highly susceptible to their environments, including the sensitivity and responsiveness of their caregivers [1]. The World Health Organization (WHO), United Nations Children’s Fund (UNICEF) and World Bank Nurturing Care Framework for Early Childhood Development was launched in 2018. It is underpinned by evidence of the strong links between maternal, newborn and child health, and identifies poor caregiver mental health as a major risk to optimal early childhood development in resource-constrained settings [1, 2].

Non-psychotic mental health problems, including depression, anxiety, somatoform and adjustment disorders are difficult to distinguish in primary care, and Goldberg and Huxley proposed that they therefore be grouped and called common mental disorders (CMDs) [3]. Women who experience mental health problems during pregnancy (antenatal CMDs) are less likely to attend antenatal care or adhere to health promotion recommendations, and more likely to experience substance misuse and lower than expected weight gain [4]. Antenatal depressive symptoms are associated not only with adverse obstetric outcomes such as intrauterine growth restriction, preterm birth and low birth weight, but also with poor early childhood development [4, 5]. If undetected and unassisted, poor antenatal mental health can become a risk factor for postnatal or chronic mental health problems [6].

In high-income countries (HIC), approximately 10% of pregnant women experience antenatal depression, whereas in low- and middle-income countries (LMIC), the weighted mean prevalence of antenatal CMDs is significantly higher (15.6%, 95%CI:15.4–15.9%) [7, 8]. There is a double disparity. In addition to the disproportionate burden of antenatal CMDS among women in LMICs, there are serious imbalances between LMICs and HICs in availability of local data on which to base policies and programs. The first systematic review of the evidence found that only nine of the then 112 low and lower-middle income countries had prevalence data about antenatal CMDs [8]. Nevertheless, the wide prevalence range (5.2–32.9%) revealed a gradient with the highest prevalence being among the poorest women with the least access to services.

The WHO Commission on the Social Determinants of Health concluded that disparities in health status within and between nations were attributable to social circumstances [9]. The WHO Conceptual Framework for the Social Determinants of Health provides a mechanism to investigate and comprehend these disparities [10]. It considers both structural (e.g. social and economic policies and cultural norms) and intermediary (e.g. socioeconomic position, living conditions and quality of relationships) determinants [10]. Most investigations of risks for antenatal CMDs have focused on the intermediary determinants. Women occupying a low socioeconomic position, living in crowded or inadequate housing, or experiencing violence perpetrated by an intimate partner, coincidental adverse life events, food insecurity, reproductive health problems or past mental health problems, or who lack social support are at increased risk of antenatal CMDs [4, 8, 11–13].

There is however, little evidence available about structural determinants of antenatal CMDs. Lund et al. [14] reviewed the evidence about links between poverty and CMDs, and concluded that people living in poverty are at increased risk of mental health problems because of stress, difficult life events, social exclusion and limited access to health care. These findings favoured the social causation hypothesis, where adverse economic conditions lead to mental health problems, as opposed to the social drift hypothesis, where people with poor mental health fall into poverty. The review did not however, disaggregate data for specific life stages, such as women who are pregnant.

Vietnam has undergone rapid economic and social development in recent decades [15]. Since 2000 we have generated epidemiological evidence about the mental health of women in rural Vietnam. Four rigorous studies, reported in six papers, examined the prevalence and intermediary determinants, but not the socioeconomic and political context, of antenatal CMDs [16–21].

Evidence is lacking about how structural determinants, such as the socioeconomic and political context, can influence antenatal mental health, both in Vietnam, and internationally. It is valuable for all countries to understand how the socioeconomic and political context interacts with the mental health of pregnant women. By understanding the structural determinants in detail, countries may develop other ways to support and improve antenatal mental health beyond health service provision. Thus, the aim of this study was to investigate whether changes in the economic and social context in Vietnam, were linked to population-level indicators of antenatal CMDs experienced by women.

## Methods

Secondary analyses of data from two epidemiological studies conducted in 2006 and 2010 in Ha Nam Province, Vietnam (the original studies). These studies collected data on intermediary and some structural determinants. These studies are described briefly below, and in detail in their respective peer-reviewed publications [17, 18]. In addition, for the current study, we searched for policy changes in Vietnam, to inform the other structural determinants.

### Setting

Vietnam, a country in South East Asia, was reclassified from a low- to a lower-middle income country in 2009 [22]. In 2006, Vietnam had a population of 85 million people, and 72% were living in a rural area [23, 24]. In 2010, the population was 88 million people, and 70% were residing in a rural area [23, 24]. Ha Nam is a province in the Red River Delta region in northern Vietnam, with a population of 880,000 people.

### Current study: policy mapping

In 1986, the Vietnamese government initiated political and economic reform [15], implemented through policies and programs to reduce poverty and improve social conditions and thereby promote socioeconomic development. We identified laws, policies and programs introduced since 1986 that may have contributed to the socioeconomic and political context.

### Original study: 2006

The aim of this cross-sectional study was to establish the prevalence and determinants of CMDs experienced during pregnancy or the postpartum period, among women living in a rural and an urban province [18]. Only data from the women who were pregnant and living in the rural province were included in the secondary analyses.

### Original study: 2010

The original study was a prospective cohort study which aimed to examine the relationship between antenatal CMDs and maternal nutrition, and subsequent infant health and development [17]. Women were assessed in early (12–20 weeks gestation) and late (more than 28 weeks gestation) pregnancy and, with their babies, at one week and 26 weeks postpartum. In this secondary analysis, we included sociodemographic characteristics (early gestation interview) and data about women’s mental health and other psycho-social factors (late gestation interview).

### Original studies: participant recruitment

In each study, an independent statistician randomly selected communes from a list of all communes in Ha Nam. In the selected communes, announcements about the studies were made over village loudspeaker systems, notes were written on blackboards at commune health stations, and commune health workers visited the households of women whose pregnancies met gestational inclusion criteria. Women who were interested in participating voluntarily attended the commune health station on the day that the research team visited.

### Original studies: data collection procedure

In rural Vietnam, people were not familiar with completing self-report questionnaires, so all data were collected in structured individual face-to-face interviews, conducted by locally trained and experienced health researchers from the Research and Training Centre for Community Development in Hanoi. Interviews were conducted in private rooms at commune health centres, or occasionally in women’s homes.

### Original studies: data collection tools

Data collection tools are described in Table 1.

**Table 1:** Data collection tools used in the two original studies

Variable	Measure
Symptoms of antenatal common mental disorders (CMDs)	- Edinburgh Postnatal Depression Scale – Vietnam validation (EPDS-V) [25] - Ten items each scored on a Likert scale from 0 to 3, total score ranges from 0 to 30 - Translated, culturally verified and formally validated in Vietnam, against psychiatrist-administered structured clinical interviews, cut-off score of 3/4 [26]
Mental health history	- Study-specific question: history of mental health problems
Intimate relationships	- Intimate Bonds Measure – Vietnam validation (IBM-V) [27] - Perceived aspects of an intimate relationship: care and control (two subscales) - 24 items each scored on a Likert scale from 0 to 3, scores from the 12 questions in each subscale added together to produce subscale scores - Translated, culturally verified and formally validated in Vietnam, against local indicators of quality of intimate relationships, reports of emotional and physical intimate partner abuse and indictors of CMDs [28]
Experiences of interpersonal violence	- Study-specific questions: fear of family members
Household Wealth Index	- Following World Bank Method [29] - Questions assessing roof material, floor material, type of latrine, cooking material and household assets (motorbike, coloured TV, video or DVD player, landline or mobile phone, refrigerator, air conditioner, water heater, washing machine and computer)
Poor household status	- Study-specific question: recognition as a poor household by the government
Reproductive history	- Study-specific questions: age at first pregnancy, gravidity, parity, abortion history and previous child deaths
Current pregnancy	- Study-specific questions: knowledge of foetal sex, whether or not the pregnancy was welcome, illness during pregnancy, use of recommended iodised salt and iron supplements and breastfeeding intentions
Demographic characteristics	- Age, education, occupation, income and marital status
Current life adversity	- Study-specific question: current stressful or adverse life events

### Current study: data management and statistical analyses

Household Wealth Index was calculated using the World Bank Method [29]. Household Wealth Index quartiles were dichotomised, with the lowest quartile (poorest) in one category, and the top three quartiles in the other category. Total EPDS-V score was treated as a continuous variable, where a higher score indicates poorer mental health, and the mean score was calculated for each study year. Mean subscale scores were calculated for the IBM-V subscales in each study year. Other categorical variables used in the model were reduced to binaries.

The analyses were conducted in two stages. In the first stage, descriptive analyses were conducted for each of the two datasets. Bivariable chi-squared and independent t-tests were conducted to assess whether socioeconomic characteristics and EPDS-V scores differed between 2006 and 2010. In the second stage, we used mediation analyses. Originally described by Wright [30] and popularised by Baron and Kenny [31], mediation analysis examines whether the relationship between an independent variable and a dependent variable is influenced by a third variable, the mediator. This type of analysis, commonly used in social and cognitive psychology, enables a deeper understanding of how an effect occurs [32].

**Fig. 1:**
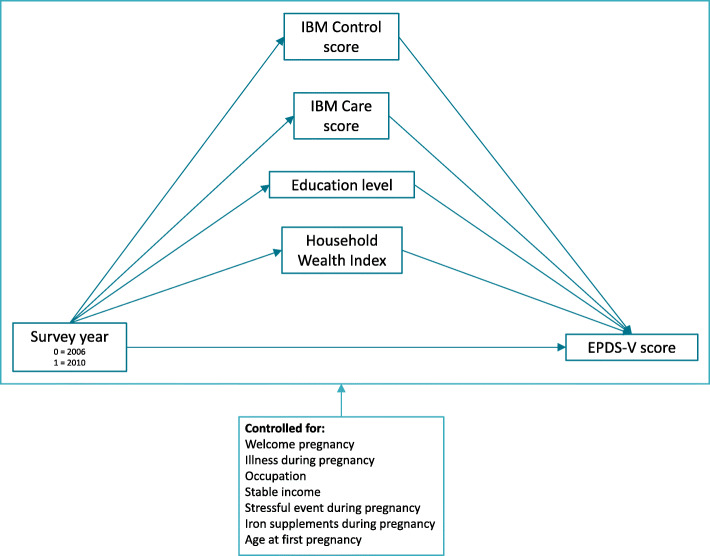
Path analysis model. IBM – Intimate Bond Measure, EPDS-V – Vietnam-validated Edinburgh Postnatal Depression Scale

In this study, mediation analyses were used to establish whether indicators of social and economic development mediated the difference in antenatal mental health (EPDS-V score) (dependent variable) between the two study years (independent variable). This mediation analysis was conducted using path analysis in Mplus Version 7.4 [33]. Missing data were handled using full information maximum likelihood (FIML) estimation under the assumption of missing at random. Four potential mediators were tested simultaneously in Mplus. These analyses enabled us to calculate the total effect of survey year on EPDS-V score, the direct effect (when controlling for mediators), the total indirect effect via all mediators, and specific indirect effects via each mediator, whilst controlling for some variables (see Fig. 1).

### Ethics approval

The 2006 Study was approved by the University of Melbourne’s Human Research Ethics Committee (HREC No. 050793), and the Vietnam Medical Association’s Scientific Committee (107/THYH). The 2010 Study was approved by the University of Melbourne’s Health Sciences Human Ethics Committee (Ethics ID 0932624), the Vietnam Medical Association Ethics and Scientific Committee, and the Ha Nam Provincial Health Department Ethics Committee. Ethics approval for this study was obtained from Monash University’s Human Research Ethics Committee (Project ID 19406).

## Results

**Fig. 2:**
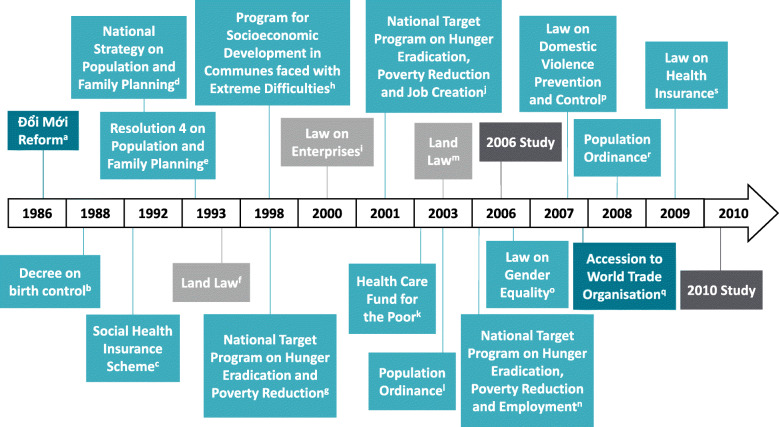
Timeline showing a series of laws, policies and programs introduced in Vietnam (1986–2010). Dark green - macroeconomic policies, light green - public policies, light grey - social policies, dark grey - two study years analysed*.* a. Political and economic reform [15]. b. Couples were encouraged to have maximum two children, by delaying childbearing or spacing births [34]. c. Nation-wide mandatory health insurance scheme covering the formally employed, civil servants, staff of international representative organisations, pensioners and people with disabilities [35]. d. National Strategy to reduce the total fertility rate [34]. e. Two-child policy introduced, but not legislated [34]. f. Households gained the right to transfer, exchange, inherit, rent and mortgage their land [36]. g. Program to reduce the proportion of poor households and eliminate chronic hunger [37]. h. Program for socioeconomic development targeted at ethnic minorities and people living in mountainous or remote areas [37]. i. Individuals were permitted to set up and run a business without major input from the government [15]. j. Program to reduce poverty, improve access to basic infrastructure, and reduce the unemployment rate [37]. k. Fund that supports access to health care for people in the lowest socioeconomic positions and ethnic minorities [35]. l. Population ordinance allowing people to decide the number of children that they have, birth timing and spacing [34]. m. Mandate that the certificate of land use lists the names of both the husband and wife [38]. n. Program to reduce poverty, improve access to basic infrastructure, and reduce the unemployment rate [39]. o. Law that defines and promotes gender equality, acknowledges the responsibilities of agencies and considers violations [40]. p. Law that makes domestic violence a crime, outlines the duties required to prevent and control domestic violence and protect victims, and outlines the consequences for domestic violence perpetrators [40]. q. Vietnam welcomed into the World Trade Organization [15]. r. Two-child policy re-introduced [34]. s. Health insurance made compulsory for children under six, the poor and near-poor, and the elderly [35]

A series of laws, policies and programs introduced in Vietnam since 1986 are shown in Fig. 2. Notably, to our knowledge, no changes were made to health systems or services related to mental health during the interval between 2006 and 2010.

### Structural and intermediary determinants

Data were contributed by 134 pregnant women from the 2006 study and 419 pregnant women from the 2010 study (total *n* = 553, recruitment fraction: 91% (2006), 84% (2010)).

The characteristics of participants are displayed in Table 2. Compared to 2006, more women had completed secondary school (Year 9) or a higher level of education, and more women were factory, government or private sector employees. More women were living in the upper three quartiles of household wealth in 2010, and fewer women were classified as poor by the local government, compared to in 2006.

**Table 2:** Characteristics of participants in 2006 and 2010

	Number of women (%)	
	2006 (***n*** = 134)	2010 (***n*** = 419)
**Education level**		
Did not complete secondary school	49 (36.6)	78 (18.7)
Completed secondary school or higher*	85 (63.4)	240 (81.3)
**Occupation**		
Factory/government/private sector employee	16 (11.9)	106 (25.3)
Farmer/student/trader/handicraft worker	118 (88.1)	313 (74.7)
**Job provides a stable income**	103 (76.9)	291 (70.1)
**Married**	133 (99.3)	418 (99.8)
**Household wealth index**		
Lowest quartile	79 (59.0)	60 (14.3)
Upper three quartiles	55 (41.0)	359 (85.7)
**Recognised as a poor household**	17 (12.7)	17 (4.1)
**Age at first pregnancy (mean (±SD))**	21.39 (±2.89)	22.50 (±3.44)
**Number of pregnancies (mean (±SD))**	2.08 (±1.18)	2.29 (±1.24)
**Number of abortions**		
None	124 (92.5)	341 (81.4)
At least 1	10 (7.5)	78 (18.6)
**Parity**		
No children	55 (41.4)	158 (37.9)
1 child	47 (35.3)	183 (43.9)
More than 1 child	31 (23.3)	76 (18.2)
**Child death (at birth or in the first week of life)**	3 (3.8)	9 (3.4)
**Foetal sex is known by the woman during pregnancy**	95 (70.9)	386 (92.1)
**Pregnancy is welcome**	104 (77.6)	370 (88.3)
**Illness during pregnancy**	38 (28.4)	163 (38.9)
**Intending to breastfeed exclusively**	78 (58.2)	378 (90.2)
**Using iodised salt during pregnancy**	125 (93.3)	375 (89.5)
**Using iron supplements during pregnancy**	100 (74.6)	379 (90.5)
**IBM Care subscale, (mean (±SD))**	32.26 (±4.51)	31.86 (±4.37)
**IBM Control subscale, (mean (±SD))**	12.62 (±11.03)	9.45 (±5.78)
**Frightened of other family member (past year)**	7 (5.2)	27 (6.4)
**Stressful life event during pregnancy**	22 (16.4)	89 (21.2)
**History of a mental disorder**	0 (0.0)	0 (0.0)

SD – standard deviation, IBM – Intimate Bonds Measure

*In Vietnam, completion of secondary school is equivalent to Year 9 in Australia

The women in 2010 had become pregnant for the first time at an older average age than those in 2006. More women knew the sex of their foetus in 2010 compared to in 2006. There were no differences in gravidity or parity between 2006 and 2010. The number of women who had experienced at least one induced abortion was nearly three items higher in 2010. Many more women described their pregnancy as welcome in 2010. In 2006, just over half of the women were intending to breastfeed exclusively in the first six months after giving birth, whereas the majority of women were intending to breastfeed exclusively in 2010.

There was no difference in the mean IBM Care subscale score between the two study years. The IBM Control subscale score was lower in 2010 than in 2006. More women reported a stressful life event during pregnancy in 2010 than in 2006. No women in either study year had been previously diagnosed with or received care for a mental disorder.

### Indicator of antenatal common mental disorders

The mean EPDS-V score was higher in 2006 (3.82 ± 4.46) compared to in 2010 (2.75 ± 4.18, *p* = 0.012).

### Mediation analysis

The total, direct and indirect effects of survey year on EPDS-V score are shown in Table 3 (full path analysis model in Appendix Table 4). The significant negative coefficient for the total effect indicates that mean EPDS-V score was lower in 2010 than in 2006. The direct effect when controlling for the mediators was not significant, and the total indirect effect via all mediators was significant, which indicates that the relationship between survey year and EPDS-V score was mediated by at least one of the indicators of socioeconomic development tested as mediators. The specific indirect effect coefficients revealed that two factors significantly mediated the relationship between survey year and EPDS-V score: HWI and IBM Control. Education level and IBM Care did not mediate the relationship.

**Table 3:** Effect of survey year (2010 vs. 2006) on EPDS-V score, generated from path analysis

	Regression coefficient	95% CI
Total effect	−1.11	− 1.92, − 0.30
Direct effect	−0.17	− 1.14, 0.80
Total indirect effect	−0.94	− 1.60, − 0.29
Specific indirect effects		
Via HWI	−0.79	−1.30, − 0.28
Via Education level	0.13	−0.10, 0.37
Via IBM Care	0.08	−0.06, 0.21
Via IBM Control	−0.36	−0.56, − 0.16

EPDS-V – Edinburgh Postnatal Depression Scale (Vietnam validation), HWI – Household Wealth Index, IBM – Intimate Bond Measure

## Discussion

This study is, to our knowledge, the first to provide evidence from a lower-middle income country of the association between national policies for socioeconomic development and population-level indicators of women’s antenatal mental health.

A strength of this study is that it drew on data from two rigorous community-based investigations. The 2006 and 2010 studies used identical data sources and recruitment and data collection procedures, and the same health researchers were involved in data collection. Participants were recruited from randomly-selected communes, and the recruitment fractions were high, indicating accurate representation of the population. We were able to examine differences in population-level characteristics, because the same data sources and procedures were used to collect data from independent groups of women in the same setting four years apart.

There were some limitations of this research. As we completed secondary analyses of existing data, we were limited to the original data collected. Some characteristics that were only collected in the 2010 study [17] (e.g. food security, number of antenatal care visits, experiences of specific violent behaviours perpetrated by an intimate partner or other family member) could not be included in the analyses, and may have influenced the findings. Further, the data were collected at least ten years ago, and may not be reflective of the current situation in Vietnam. Given that to our knowledge there are no data of this kind world wide, it remains valuable to utilise these existing data, and consider the findings in the current context. We acknowledge that the policy mapping was limited to documents published in English, and that we were not able to access some government and official documents as they are not publicly available. Additionally, the sample sizes in 2006 and 2010 were not equal (134 and 419 women, respectively). This limitation was addressed by including all participants into the analyses, and stratifying results by survey year. Finally, we acknowledge that women from only one province of Vietnam were included. This may limit the generalisability of our findings to all of Vietnam. However, efforts were made in the original studies to recruit women from randomly selected communes, to maximise the representativeness of the sample. Overall, we believe that the strengths outweigh the limitations and that the findings can be generalised with considerable confidence to other settings.

### Structural determinants and antenatal mental health

Our findings demonstrate that in Vietnam, greater household wealth and experiencing fewer controlling behaviours from an intimate partner mediated the improved antenatal mental health in 2010, compared to in 2006. These findings provide unique evidence to support the strength of the structural determinants of mental health. Whilst we could not test the relationship between the introduction of laws and policies and antenatal CMDs directly, policy mapping allows us to understand and interpret changes in the socioeconomic and political context and to investigate their associations with population health indicators.

Lund et al. [14] found that indicators of poverty: limited education, food insecurity, inadequate housing, socioeconomic position and financial stress were consistently associated in cross-sectional analyses with CMDs, but did not examine the impact on antenatal mental health problems or of transitions into or out of poverty. Our findings build on the work of Lund et al. [14] and support the social causation hypothesis, by providing evidence that poverty alleviation is associated with improvements in population-level antenatal mental health. In Vietnam, poverty reduction policies included giving poor families access to low- or no-interest bank loans, permitting families to buy land for farming and providing them with training in how to run a successful agricultural business and improve their economic status. Their children were given free or subsidised education. Privatisation of the agricultural sector and growth in the manufacturing sector provided opportunities for income-generating work outside subsistence agriculture [15]. In our data, more women in 2010 were living in households with greater wealth, had completed a higher level of education, and were working in a salaried occupation compared to in 2006. We speculate that the public policies may have altered the socioeconomic and political milieu in Vietnam, creating better living circumstances. Our data indicate that less poverty and better living circumstances may be a lever for improving antenatal mental health.

Psychological violence includes controlling and coercive behaviours and criticism. Women reported experiencing significantly lower levels of these behaviours perpetrated by their intimate partners in 2010 than in 2006, and this was linked to lower levels of symptoms of antenatal CMDs. Antenatal mental health is protected if the intimate partner relationship is experienced as affectionate, kind and trustworthy, but diminished by controlling behaviours and other forms of abuse [8]. Our findings suggest that a cultural change in gender equality may be occurring in Vietnam. In traditional Vietnamese culture, relationships are often dictated by gendered family roles, and violence against women can occur [40]. In Vietnam, a law on gender equality and a law criminalising domestic violence were introduced in 2006 and 2007, respectively, to improve gender equality and empowerment. The national laws were implemented locally through a domestic violence prevention program (personal communication with the Ha Nam Provincial Women’s Union office, 2019). This program, introduced in 70 communes in Ha Nam, involved education about domestic violence, and prevention and protection strategies for men and women. We postulate that this cultural shift may enable women to be more empowered, and men to be more considerate of the impact of their behaviours and the benefits of respectful relationships.

Reproductive autonomy for women is conceptualised as an individual woman having the power to make decisions relating to contraceptive use, whether or not she wants to have children, and the timing and spacing of pregnancies [41]. Reproductive autonomy and its influence on antenatal mental health could not be measured in this study. However, reproductive autonomy may be improved by greater empowerment and independence. The larger proportion of women with higher education and a salaried occupation, and the lower level of perceived control from intimate partners suggests that in 2010 women in Ha Nam were living in more economically advantaged circumstances and experiencing greater empowerment than they had in 2006. As a result, we speculate that reproductive autonomy, indicated by an older average age at first pregnancy, a higher proportion having accessed abortion and a higher proportion of pregnancies being experienced as welcome, may have influenced mental health during the index pregnancy.

Overall, these data indicate that the introduction of laws and policies shaped the structural determinants of health in Vietnam. They provide evidence that the socioeconomic and political context is highly relevant to the antenatal mental health of women and is potentially modifiable in ways that decrease risk and burden.

### Implications

Our findings support calls made in the WHO’s Mental Health Gap Action Programme (mhGAP) [42]. In this action plan, the need for intersectoral collaboration, both at a national level to expand mental health service delivery, and at an individual level to improve mental health is recommended. Our study provides evidence that action in non-health sectors influences mental health, specifically of pregnant women. This aligns with the United Nations’ (UN) ‘Delivering as One’ approach, which encourages countries to streamline the activities of diverse UN agencies into a more coherent and efficient collective system [43]. The findings are relevant for policy-makers globally, and indicate that mental health, particularly among sub-populations such as pregnant women should be measured as an indicator of policy impact.

Our findings also provide implications for future research, both in Vietnam and internationally. In Vietnam, the relationship between the socioeconomic and political context and antenatal mental health needs updated and continual monitoring. It is important to investigate whether the findings from this study are still evident in the current context, or if the influence of the structural determinants has changed. This research could also be replicated in other countries. In particular, it would be interesting for countries that have undergone, or are undergoing, similar rapid socioeconomic development, to assess the impact on antenatal mental health with new or existing data.

## Conclusion

Our study demonstrates how changes in the socioeconomic and political context, particularly involving poverty reduction and intimate partner relationships, can influence antenatal mental health at a population level. By improving the circumstances in which women live, we can improve their mental health during pregnancy, with flow on benefits for the nurturing care provided to their children.

## Data Availability

The datasets used and/or analysed during the current study are available from the corresponding author on reasonable request.

## Appendix

**Table 4:** Full path analysis model

**Regression models: potential mediators**		
	**Regression coefficient**	**95% confidence interval**
Household wealth index on:		
Survey year (0: 2006, 1: 2010)	-1.247	-1.520, -0.974
Occupation (0: factory, government or private sector employee, 1: farmer, student, trader, handicraft worker)	0.585	0.213, 0.957
Age at first pregnancy (years)	0.036	0.001, 0.072
Pregnancy is welcome (0: welcome, 1: not welcome)	0.303	-0.054, 0.660
Illness during pregnancy (0: no, 1: yes)	0.067	-0.211, 0.344
Stressful events (0: no, 1: yes)	0.036	-0.294, 0.365
Iron supplementation (0: yes, 1: no)	0.263	-0.096, 0.622
Education level on:		
Survey year (0: 2006, 1: 2010)	-0.475	-0.750, -0.199
Occupation (0: factory, government or private sector employee, 1: farmer, student, trader, handicraft worker)	0.916	0.517, 1.314
Age at first pregnancy (years)	0.004	-0.029. 0.038
Pregnancy is welcome (0: welcome, 1: not welcome)	0.376	0.047, 0.705
Illness during pregnancy (0: no, 1: yes)	0.107	-0.150, 0.365
Stressful events (0: no, 1: yes)	0.010	-0.325, 0.346
Iron supplementation (0: yes, 1: no)	-0.160	-0.530, 0.210
IBM Care on:		
Survey year (0: 2006, 1: 2010)	-0.516	-1.425, 0.393
Occupation (0: factory, government or private sector employee, 1: farmer, student, trader, handicraft worker)	-0.152	-1.055, 0.751
Age at first pregnancy (years)	0.020	-0.104, 0.144
Pregnancy is welcome (0: welcome, 1: not welcome)	0.152	-0.952, 1.257
Illness during pregnancy (0: no, 1: yes)	-0.638	-1.385, 0.108
Stressful events (0: no, 1: yes)	-2.429	-3.176, -1.681
Iron supplementation (0: yes, 1: no)	-1.662	-2.581, -0.742
IBM Control on:		
Survey year (0: 2006, 1: 2010)	-3.075	-4.742, -1.408
Occupation (0: factory, government or private sector employee, 1: farmer, student, trader, handicraft worker)	0.861	-1.654, 3.377
Age at first pregnancy (years)	-0.035	-0.300, 0.231
Pregnancy is welcome (0: welcome, 1: not welcome)	-0.514	-2.618, 1.590
Illness during pregnancy (0: no, 1: yes)	-0.429	-2.108, 1.250
Stressful events (0: no, 1: yes)	3.669	1.738, 5.600
Iron supplementation (0: yes, 1: no)	0.689	-1.433, 2.812
**Regression model: EPDS-V score**		
	**Regression coefficient**	**95% confidence interval**
EPDS-V score on:		
Survey year (0: 2006, 1: 2010)	-0.166	-1.136, 0.803
HWI (0: upper 75%, 1: lower 25%)	0.632	0.204, 1.024
Education level (0: completed secondary school or higher, 1: did not complete secondary school)	-0.282	-0.751, 0.187
IBM Care score	-0.147	-0.201, -0.092
IBM Control score	0.118	0.100, 0.135
Occupation (0: factory, government or private sector employee, 1: farmer, student, trader, handicraft worker)	-0.500	-1.522, 0.521
Age at first pregnancy (years)	-0.078	-0.186, 0.031
Pregnancy is welcome (0: welcome, 1: not welcome)	1.079	0.270, 1.889
Illness during pregnancy (0: no, 1: yes)	0.416	-0.252, 1.085
Stressful events (0: no, 1: yes)	3.573	2.870, 4.276
Iron supplementation (0: yes, 1: no)	-0.072	-0.924, 0.779

*IBM* Intimate Bonds Measure, *EPDS-V* Edinburgh Postnatal Depression Scale (Vietnam validation), *HWI* Household Wealth Index

## Abbreviations

CMD: Common mental disorder
EPDS-V: Edinburgh Postnatal Depression Scale – Vietnam validation
HIC: High-income countries
IBM-V: Intimate Bonds Measure – Vietnam validation
LMIC: Low- and middle-income countries
UN: United Nations
UNICEF: United Nations Children’s Fund
WHO: World Health Organization
